# Metabolic Engineering of Extremophilic Bacterium *Deinococcus radiodurans* for the Production of the Novel Carotenoid Deinoxanthin

**DOI:** 10.3390/microorganisms9010044

**Published:** 2020-12-25

**Authors:** Sun-Wook Jeong, Jun-Ho Kim, Ji-Woong Kim, Chae Yeon Kim, Su Young Kim, Yong Jun Choi

**Affiliations:** 1 School of Environmental Engineering, University of Seoul, Seoul 02504, Korea; jeongsunwook@gmail.com; 2 Material Sciences Research Institute, LABIO Co., Ltd., Seoul 08501, Korea; msri06@labio.kr (J.-H.K.); msri12@labio.kr (J.-W.K.); msri02@labio.kr (C.Y.K.); msri01@labio.kr (S.Y.K.)

**Keywords:** *Deinococcus radiodurans*, metabolic engineering, deinoxanthin, xanthophylls, antioxidation

## Abstract

Deinoxanthin, a xanthophyll derived from *Deinococcus* species, is a unique organic compound that provides greater antioxidant effects compared to other carotenoids due to its superior scavenging activity against singlet oxygen and hydrogen peroxide. Therefore, it has attracted significant attention as a next-generation organic compound that has great potential as a natural ingredient in a food supplements. Although the microbial identification of deinoxanthin has been identified, mass production has not yet been achieved. Here, we report, for the first time, the development of an engineered extremophilic microorganism, *Deinococcus radiodurans* strain R1, that is capable of producing deinoxanthin through rational metabolic engineering and process optimization. The genes *crtB* and *dxs* were first introduced into the genome to reinforce the metabolic flux towards deinoxanthin. The optimal temperature was then identified through a comparative analysis of the mRNA expression of the two genes, while the carbon source was further optimized to increase deinoxanthin production. The final engineered *D. radiodurans* strain R1 was able to produce 394 ± 17.6 mg/L (102 ± 11.1 mg/g DCW) of deinoxanthin with a yield of 40.4 ± 1.2 mg/g sucrose and a productivity of 8.4 ± 0.2 mg/L/h from 10 g/L of sucrose. The final engineered strain and the strategies developed in the present study can act as the foundation for the industrial application of extremophilic microorganisms.

## 1. Introduction

Xanthophylls are oxygenated carotenoids that have received significant interest as an ingredient in natural foods due to their association with beauty and health. In particular, xanthophylls have distinctive structural features such as terminal hydroxy groups, ketones, aldehyde, and epoxy groups that provide superior radical scavenging activity compared with carotenes such as β-carotene and lycopene [1,2,3]. Over the last few decades, significant research progress has been made on the microbial production of xanthophylls using microalgae, yeast, and other microorganisms. For example, the biosynthetic pathway for astaxanthin, zeaxanthin, and lutein, which are widely used as dietary supplements, has been discovered in various microorganisms [4,5,6]. Recently, (3R)-saproxanthin and (3R,2′S)-myxol, which enhance lipid peroxidation activity compared with β-carotene, were isolated from the marine bacterial strains 04OKA-13-27 and YM6-073, respectively [7]. Another study also reported the isolation of siphonaxanthin, which is considered a promising anti-cancer compound, from green algae within the order Siphonales [8].

Recently, extremophilic microorganisms have been recognized as a promising bioresource for the production of various xanthophylls. This is because they have evolved to produce their own xanthophylls that are involved in multiple cellular functions essential for their survival in extreme habitats. For example, 2′-isopentenylsaproxanthin was first discovered in the alkaliphilic bacterium *Jejuia pallidilutea* strain 11shimoA1 [9]. The levels of 2′-isopentenylsaproxanthin have been found to increase under high alkaline conditions (pH 9.2), suggesting that 2′-isopentenylsaproxanthin plays a crucial role in resisting extreme conditions. Another study also reported the isolation of thermozeaxanthin from the thermophilic bacterium *Thermus filiformis*, which is responsible for adaption to extremely high temperatures by stabilizing the cellular membrane [10].

*Deinococcus radiodurans* strain R1 (reclassified from *Micrococcus radiodurans*) is a radiation-resistant extremophilic microorganism that has great potential as a microbial host for the production of novel carotenoids [11]. Recently, metabolically engineered *D. radiodurans* R1 capable of producing phytoene, a colorless carotenoid, was reported for the first time [12]. The high microbial production of lycopene has also been successfully achieved via systematic metabolic engineering approaches in combination with an optimized abiotic environment, such as the presence of UV-C irradiation and conditional nutrient supplementation [13,14]. In particular, deinoxanthin ((2R)-2,1′-dihydroxy-3′,4′-didehydro-1′,2′-dihydro-β,Ψ-caroten-4-one) is a unique xanthophyll that can only be synthesized by *Deinococcus* microorganisms and is possibly associated with the ability to survive under extremely high irradiation [11,15,16]. It has also demonstrated a higher scavenging activity against singlet oxygen and hydrogen peroxide (H_2_O_2_) than other xanthophyll carotenoids such as lutein and zeaxanthin [17,18,19]. In a previous comparative study on various xanthophyll structures, deinoxanthin was shown to possess a unique structural feature consisting of extended conjugated double-bonds, a conjugated keto-group, a C-1′ hydroxyl group, and an additional C-2 hydroxyl group on the β-ring end, which may be responsible for its superior antioxidation activity [16]. Despite the significant potential of deinoxanthin as a beneficial natural compound, only molecular-level research, including the optimization of culture conditions and carbon sources, has been conducted to date [15,20,21,22,23,24], and no studies have been reported on its mass production via systematic metabolic engineering. Thus, the production of deinoxanthin via systematic metabolic engineering of *D. radiodurans* R1 remains an important goal. In the present study, we report, for the first time, the engineering of the carotenoid biosynthetic pathway in *D. radiodurans* R1 to produce large amounts of deinoxanthin.

## 2. Materials and Methods

### 2.1. Strains, Plasmids, and Culture Medium

The strains and plasmids used in the present study are listed in Table 1. *Deinococcus radiodurans* R1 (ATCC13939) and its derivatives were cultivated in TGY media (0.5% trytone, 0.1% glucose, and 0.3% yeast extract). Batch culturing for the production of deinoxanthin was conducted in modified TGY medium (per liter, pH 7.0) containing 5 g/L tryptone, 5 g/L yeast extract, 10 g/L glucose, 0.5 g/L MgSO_4_∙7H_2_O, and 1 mg/L MnCl_2_. *Escherichia coli* DH5α was used as the host strain for the construction of recombinant plasmids and cultured at 37 °C in Luria-Bertani (LB) medium. When necessary, antibiotics were added at a final concentration of 100 μg/mL ampicillin for *E. coli*, and 3 μg/mL chloramphenicol and 25 μg/mL kanamycin for *D. radiodurans*.

### 2.2. DNA Manipulation and Plasmid Construction

The primer sets used for plasmid construction are listed in Table S1. All recombinant plasmids and PCR products were purified using the MegaQuick-Spin plus fragment DNA purification kit (Intron Biotechnology, Seongnam, Korea) and the NucleoSpin kit (Macherey-Nagel GmbH & Co., Dueren, Germany). The recombinant plasmids pAM41, pAM73, and pAM104 were obtained from a previous study (Table 1) [12]. The pAM104 plasmid was directly transformed into *D. radiodurans* following a standard protocol [12], and the transformants were screened on 2 × TGY-chloramphencol agar plates. The resulting *D. radiodurans* strain expressing *dxs* and *crtB* was designated as DX1.

The endogenous genes *crtB* and *dxs* under the *groE* promoter were integrated into the *D. radiodurans* chromosome CP1 between the *drC0004* and *drc0007* regions using a Cre-*loxP* system [25]. The gene integration process is depicted in Figure S1. To integrate *crtB* into the *D. radiodurans* genome, approximately 1 kb of DNA fragments containing partial sequences of *drC0006* and *drC0007* was amplified via PCR using appropriate primer sets. The resulting PCR fragments were assembled using fusion PCR and ligated with pTOP Blunt V2 plasmid (Enzynomics) (designated as pdrc6). The DNA fragment containing *crtB* and the *groE* promoter was amplified using groE-F1/crtB-R1 primers and the pAM41 plasmid as a DNA template. The resulting PCR product was digested with *Kpn*I/*EcoR*V and ligated into pAM1. The DNA fragment containing the *groE* promoter, *crtB,* and the kanamycin resistance cassette flanked with two *loxP* sequences was amplified using a groE-F2/crtB-R2 primer set. The resulting PCR product was ligated into the pdrc6 plasmid at the *Stu*I site (referred to as pdrc6-crtB, Table 1). An integrative plasmid for the insertion of *dxs* was also constructed. Approximately 1 kb of upstream/downstream DNA fragments containing *drC0004* and *drc0005* was amplified using drc04-1/drc04-2 and drc05-1/drc05-2 primer sets, respectively. The PCR fragments were ligated into the pAM1 plasmid at the *Kpn*I/*Pst*I and *BamH*I/*Sph*I sites (designated as pdrc4). The DNA fragment containing *dxs* and the *groE* promoter was amplified using dxs-F1/dxs-R1 primers and the pAM73 plasmid as a DNA template. The resulting PCR product was digested with *Pst*I/*EcoR*V and ligated with pdrc4 in the same way as for the restriction enzyme sites (referred to as pdrc4-dxs, Table 1). The integrated plasmids pdrc4-dxs and pdrc6-crtB were sequentially transformed into *D. radiodurans*, and the resulting transformants were screened using a 2 × TGY-kanamycin agar plate. To remove the kanamycin resistance gene from the *D. radiodurans* genome, pAM2, which expresses Cre recombinase, was transformed and completely cured in *D. radiodurans* following a previously described method [25]. Gene integration and the selective removal of markers in the *dxs*- and *crtB*-integrated *D. radiodurans* mutants were confirmed using plating assays, diagnostic PCR using appropriate primer sets, and DNA sequencing (Figure S2). This final strain was designated as *D. radiodurans* DX2 and used for deinoxanthin production.

### 2.3. Shake Flask Cultivation

To examine deinoxanthin production in the engineered *D. radiodurans* strains, fresh colonies were inoculated into 3 mL of TGY medium and cultivated overnight (16–20 h) at 30 °C and 200 rpm to produce an OD_600_ of 1.8–2.1. Seed cultures (1%) were then transferred to a 250 mL baffled flask with 50 mL of modified TGY medium and cultivated at 30 °C and 200 rpm for 48 h. To examine the effects of temperature and the carbon source on cell growth and deinoxanthin production, the *D. radiodurans* strain DX2 was initially cultivated in 50 mL of modified TGY medium at 30 °C for 24 h and then grown at 30, 32, or 37 °C for 48 h after the change in temperature. Glucose, sucrose, and fructose were used as a carbon source (10 g/L) in modified TY medium (pH 7.0). Cell growth was monitored by measuring the absorbance at 600 nm (OD_600_) using a microplate reader (Biotek, Winooski, VT, USA). Cultured broth (10 mL) was harvested by centrifugation at 4000 rpm for 20 min and washed twice with deionized water (DW). The obtained pellets were freeze-dried and the weight of the dried cells was measured before carotenoid extraction.

### 2.4. Carotenoid Extraction and Analytical Methods

Total carotenoids from the pellets were extracted using 1 mL of methanol and sonication for 1 min, which was repeated twice. To produce standard deinoxanthin, 1 L of wild-type *D. radiodurans* cells grown for 48 h at 30 °C was harvested at 4000 rpm for 30 min. After washing twice with DW, deinoxanthin was extracted from the pellets with 50 mL of methanol. The deinoxanthin extracts were concentrated using a rotary evaporator (IKA, Seoul, Korea) at 55 °C and 70 rpm and completely lyophilized using a freeze dryer (Operon Co., Ltd., Gimpo, Korea). A deinoxanthin calibration curve (5 to 200 mg/L) was constructed to quantify the deinoxanthin levels.

Carotenoid analysis was conducted using previously described methods [12,24]. The deinoxanthin was analyzed using high-performance liquid chromatography (HPLC, Agilent 1260 InfinityII, Agilent Corp., Santa Clara, CA, USA) equipped with a Zorbax Eclipse XDB-C18 column (4.6 × 150 mm; Agilent Corp., Santa Clara, CA, USA) and a UV/VIS detector at 480 nm. A mixture of acetonitrile, methanol, and isopropanol (40:50:10, *v*/*v*/*v*) was used as the mobile phase with a flow rate of 0.8 mL min^−1^ at 40 °C. The concentration of the carbon source in the culture media was determined using HPLC equipped with a refractive index detector. Separation was conducted with a MetaCarb 87H column (4.6 × 250 mm; Agilent Corp., Santa Clara, CA, USA) at 60 °C. As the mobile phase, 0.005 N H_2_SO_4_ was used at a flow rate of 0.5 mL min^−1^. LC-MS analysis was performed using an LC/MS detection system (Agilent 6120 Quadrupole LC/MS, Agilent Corp., Santa Clara, CA, USA) to determine the molecular mass of deinoxanthin from the crude extracts. The LC/MS conditions were as follows: a drying gas temperature of 350 °C, a drying gas flow rate of 10 L min^−1^, a capillary voltage of 3.5 kV, and a nebulizer pressure of 40 psi_g_.

### 2.5. Real-Time Quantitative PCR (RT-qPCR)

Total RNA was extracted from 1 mL of *D. radiodurans* cells with RiboEX reagent (GeneAll, Seoul, Korea) according to the manufacturer’s instructions. Cell disruption was instigated by adding 500 μL of 0.1-mm diameter silica beads (Biospec, Bartlesville, OK, USA). DNA contaminants were completely removed from the crude lysates using DNase I (Takara, Tokyo, Japan) treatment and purified using an RNeasy Mini Kit (Qiagen, Hilden, Germany). For real-time quantitative PCR analysis (RT-qPCR), cDNA was synthesized from 1 μg of total RNA using a SuperiorScript III cDNA synthesis kit (Enzynomics, Daejeon, Korea) following the manufacturer’s instructions. RT-qPCR amplification was conducted using the RbTaq Fast qPCR 2X premix (Enzynomics, Daejeon, Korea) on a Bio-Rad CFX Real-Time System (Bio-Rad, Hercules, California, USA). PCR reactions were conducted using the following process: one cycle of 95 °C for 3 min, followed by 40 cycles of 95 °C for 10 s and 60 °C for 30 s. The gene *dr1343*, which encodes glyceraldehyde-3-phosphate dehydrogenase (*gap*), was chosen as an internal control to normalize the transcriptional expression of the tested target genes. Approximately 100 bp of unique sequences from the genes encoding *gap* (*dr1343)*, *groE* (*dr0606*), *crtB* (*dr0862*), and *dxs* (*dr1475*) was amplified using the following primers: dr1343F/R (5′- caacgacctgaccgacaacc-3′, 5′-ggctgctttcgtcgtactcc-3′), dr0606F/R (5′-gtcaaggaaggcgacaccgt-3′, 5′-tcgacaatggcgagcaggtc-3′), dr0862F/R (5′-caggccgtattcgtcgagca-3′, 5′-aactcggtcaggcgatgcag3′), and dr1475F/R (5′-ctgcgcgggatgctcaagta-3′, 5′-atttcaggtccggccacgtc3′).

### 2.6. DPPH Assays

The antioxidant activity of the carotenoids was determined using 2, 2-diphenyl-1-picryl-hydrazyl (DPPH, Alfa Aesar, Tewskbury, MA, USA) free radical assays as previously described [26]. Briefly, 100 μL of DPPH solution (0.2 mM) in methanol was added to 100 μL of different concentrations of carotenoid solutions and incubated for 30 min at room temperature in the dark. After the reaction, the absorbance of the samples was measured at 517 nm using a microplate reader (Biotek, Winooski, VT, USA). The following standard carotenoids were used: phytoene (TRC, North York, ON, Canada), beta-carotene (Sigma-Aldrich, St. Luis, MO, USA), lycopene (Sigma-Aldrich, St. Luis, MO, USA), and astaxanthin (Cayman, MI, USA). Ascorbic acid (TCI, Tokyo, Japan) was used as a positive control in the DPPH assays. Radical scavenging activity was calculated as a percentage of DPPH discoloration using Equation (1):

(1) DPPH scavenging activity (%) = [Acontrol− (Asample −Asample blank)/Acontrol] × 100

where A_contol_, A_sample_, and A_sample blank_ are the absorbance of the DPPH solution, the carotenoid solution with DPPH, and the carotenoid solution without DPPH, respectively.

## 3. Results

### 3.1. Construction of a Base Strain via the Genome-Based Overexpression of Rate-Limiting Steps

Previous research has identified the deinoxanthin biosynthetic pathway in *Deinococcus* strains via the comparative functional analysis of putative carotenoid genes [16,20,21,22,23]. According to a previous report, deinoxanthin biosynthesis is initiated through the methylerythritol 4-phosphate (MEP) pathway, and the carotenoid γ-carotene is utilized as a core backbone at the end of the synthesis process. During this process, unique structural features that lead to superior antioxidant performance are created via enzymatic modifications such as hydroxylation, desaturation, and ketolation mediated by 1,2-hydratase (CruF), carotenoid 3′,4′- desaturase (CrtD), carotenoid ketolase (CrtO), and carotene 2-β-hydroxlyase (DR2473) (Figure S1) [22].

**Figure 1:**
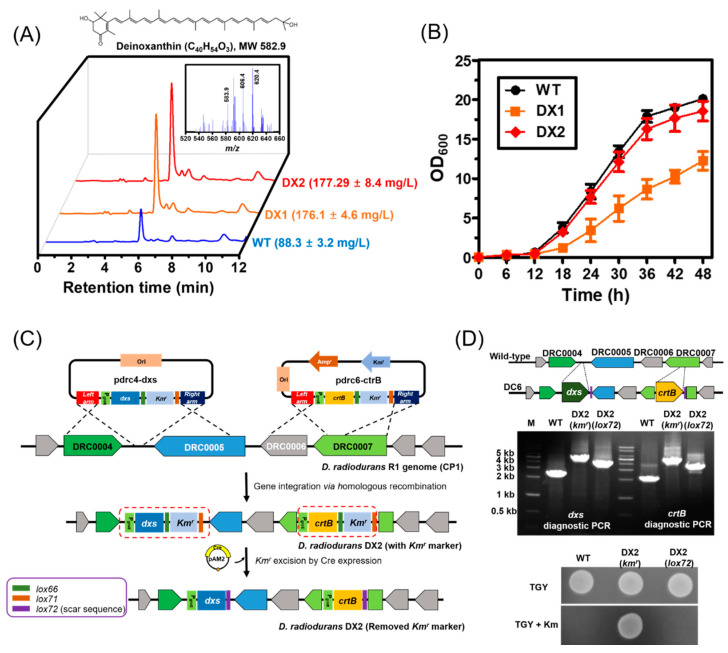
Cell growth and deinoxanthin production in engineered *D. radiodurans* strains. (**A**) Deinoxanthin production in the *D. radidurans* strains. HPLC spectra of the total carotenoids extracted from the *D. radiodurans* strains are presented. Inset presents the LC/MS spectra of total carotenoids obtained from the DX2 strain in positive ionization mode (ESI^+^). The molecular ions at *m/z* 583.9, 606.4, and 620.4 were assigned to ([M + H]^+^), ([M + Na]^+^), and ([M + K]^+^), which correspond to the presence of deinoxanthin. (**B**) Growth profiles of the plasmid-free *D. radiodurans* strain DX2 expressing the *dxs* and *crtB* genes over 48 h of cultivation. (**C**) Scheme of the construction of plasmid- and antibiotic-free *D. radiodurans* DX2 strain for deinoxanthin overproduction. The detailed procedure is described in the Materials and Method section. (**D**) Diagnostic PCR of *dxs* or *crtB* gene inserted in DX2 strain (upper panel). Plating assay of DX2 strain on trytone, glucose, and yeast (TGY) agar plate with or without kanamycin (25 μg/mL) (bottom panel). The data represent the mean and standard deviation of biological triplicates.

Previous studies have reported that *dxs*, which encodes 1-deoxy-D-xylulose-5-phosphate synthase, and *crtB*, which encodes phytoene synthase, are rate-limiting steps in the carotenoid biosynthetic pathway in *D. radiodurans*. Thus, metabolically engineered *D. radiodurans* strains that overexpress both genes have been used as the base strain for the production of various carotenoids [12,13]. Because wild-type *D. radiodurans* also contains the deinoxanthin biosynthetic pathway, *dxs* and *crtB* were overexpressed via plasmid-based overexpression to produce a deinoxanthin-overproducing strain. The resulting strain (DX1) produced 176.1 ± 4.6 mg/L of deinoxanthin, twice as much as that obtained from the wild-type strain (88.3 ± 3.2 mg/L) (Figure 1A). This demonstrates that the overexpression of *dxs* and *crtB* also occurred during deinoxanthin production in the *D. radiodurans* strain. However, the cell growth of the DX1 strain (OD_600_ of 12.5 ± 1.2) was 39% lower at the end of culturing than the wild-type (OD_600_ of 20.1 ± 0.4) (Figure 1B). This is probably due to the severe metabolic burden resulting from plasmid-based overexpression [17]. Thus, a *D. radiodurans* strain in which *dxs* and *crtB*, regulated by the *groE* promoter (P_groE_), were inserted into the genome was constructed using a plasmid-free Cre-*loxP* system (Figure 1C,D) [25]. As expected, the cellular growth of the resulting strain (DX2) was recovered while maintaining high deinoxanthin production (177.29 ± 8.4 mg/L) (Figure 1B). Therefore, the DX2 strain was selected as the base strain for improving deinoxanthin production by optimizing the culture conditions.

### 3.2. Optimization of Culture Conditions to Enhance the Production of Deinoxanthin

**Figure 2:**
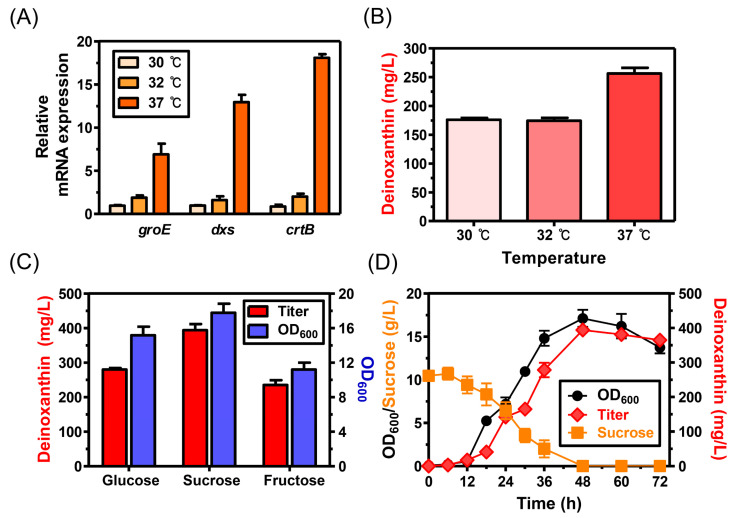
Deinoxanthin production by the *D. radiodurans* strain DX2 under different culture conditions. (**A**) Transcription profiles of the genes *groE*, *dxs*, and *crtB* based on the growth temperature. The strain DX2 cultured at 30 °C was used as a control to normalize gene expression. Each sample was taken at 36 h for qRT-PCR analysis. (**B**) Deinoxanthin production under different growth temperatures. (**C**) Deinoxanthin production using different carbon sources (10 g/L). (**D**) Shake flask batch culture in TY medium containing 10 g/L sucrose under 72 h of incubation. All experiments were performed in triplicate, and the error bars represent the standard deviation.

To increase deinoxantin production, the culture conditions were optimized, in particular the temperature and carbon source. In general, the *groE* promoter, which originates from the heat shock protein GroESL (an operon consisting of *dr0606* and *dr0607*) in *D. radiodurans* R1, is the most widely used promoter in *D. radiodurans* research due to its high stability and strong activity [18,19]. It is also known that the expression levels of genes regulated by the *groE* promoter vary depending on the temperature to which they were exposed [27]. Therefore, because *dxs* and *crtB* were regulated by the *groE* promoter, it was reasoned that deinoxanthin production can be improved by optimizing the culture temperature. In order to determine the optimal temperature for deinoxanthin production, the mRNA expression levels of *groE*, *dxs*, and *crtB* were monitored in a temperature-dependent manner. As shown in Figure 2A, a gradual increase in mRNA expression patterns was confirmed as the temperature rose. A higher than 6-fold increase in the mRNA expression levels of *groE* was observed at 37 °C compared to those obtained at 30 °C. With the use of the *groE* promoter at 37 °C, the mRNA expression levels of *dxs* and *crtB* dramatically increased 13- and 18-fold, respectively, under the same conditions. Moreover, 256.5 ± 13.8 mg/L of deinoxanthin was produced at 37 °C, which was approximately 32% higher than that obtained at 30 (176.1 ± 4.6 mg/L) and 32 °C (174.4 ± 7.3 mg/L) (Figure 2B). These results suggest that external conditions that mimic the typical habitat of extremophilic microorganisms are an important consideration when engineering them for industrial applications.

Optimal carbon sources were also investigated. According to a previous report, sucrose, which consists of glucose and fructose, is the most effective carbon source for the production of carotenoids in tomato at the metabolic level [28]. To examine if this also applies to *D. radiodurans*, we tested sucrose, monomeric glucose, and monomeric fructose as the sole carbon sources for the production of deinoxanthin. As reported in the previous study, sucrose was found to be the optimal carbon source for deinoxanthin production. The strain DX2 supplemented with sucrose produced 394 ± 17.6 mg/L of deinoxanthin, which was approximately 29.0% and 40.3% higher than that obtained from glucose (280.2 ± 4.3 mg/L) and fructose (235.3 ± 1.9 mg/L), respectively. These optimized conditions produced 102 ± 11.1 mg deinoxanthin/g DCW with a yield and productivity of 40.4 ± 1.2 mg/g sucrose and 8.4 ± 0.2 mg/L/h, respectively, from 10 g/L of sucrose (Figure 2C,D).

### 3.3. Evaluation of the Antioxidant Activity of Deinoxanthin

**Figure 3:**
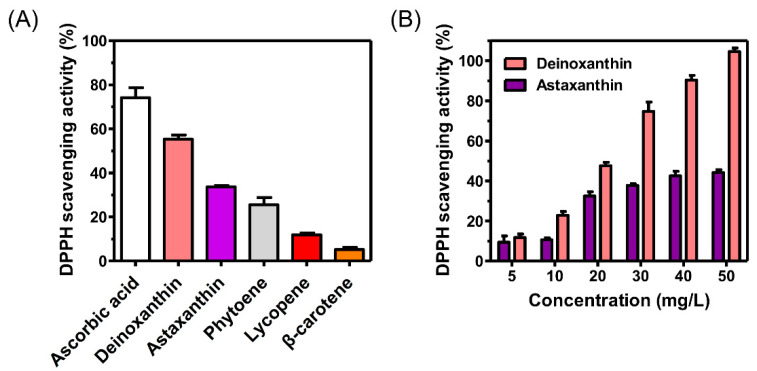
Results for 2, 2-diphenyl-1-picrylhydrazyl (DPPH) radical-scavenging assays. (**A**) Comparison of antioxidation activity with various carotenoids. Ascorbic acid (10 mg/L) was used as the positive control standard. All carotenoid concentrations were fixed at 20 mg/L for the DPPH assays. (**B**) DPPH scavenging activity of deinoxanthin and astaxanthin at different concentrations. All experiments were conducted in triplicate, and the error bars represent the standard deviation.

In order to investigate the industrial applicability and reliability of the deinoxanthin produced in the present study, in vitro DPPH (2, 2-diphenyl-1-picrylhydrazyl) radical-scavenging assays were performed. Figure 3A shows that deinoxanthin had significantly higher DPPH scavenging activity (55.3 ± 1.9%) than the carotenes phytoene (25.5 ± 3.2%), lycopene (11.9 ± 0.9%), and β-carotene (5.2 ± 1%). It also exhibited 21.7% higher DPPH radical-scavenging activity than that shown by astaxanthin (33.6 ± 1.6%). In addition, the DPPH radical-scavenging activity of astaxanthin leveled off at 40% as the concentration increased, while that of deinoxanthin gradually increased in proportion to the concentration. A 2.4-fold increase in scavenging activity was also observed for 50 mg/L deinoxanthin compared to that observed for the same concentration of astaxanthin (Figure 3B).

## 4. Conclusions

In this study, we reported the development of a genetically engineered strain of the extremophilic microorganism *D. radiodurans* that is capable of producing high levels of deinoxanthin following process optimization. Increased production of deinoxanthin was successfully achieved by integrating the genes *dxs* and *crtB* into the *D. radiodurans* genome. Deinoxanthin production was enhanced further by comparing the mRNA expression levels of the genes involved in the rate-limiting steps. It was also proven that sucrose was the optimal carbon source for deinoxanthin production. The final engineered *D. radiodurans* DX2 was able to produce 394 ± 17.6 mg/L (102 ± 11.1 mg/g DCW) of deinoxanthin from 10 g/L of sucrose under optimal conditions. Furthermore, the feasibility of the microbial production of deinoxanthin was confirmed by in vitro DPPH radical-scavenging assays. In conclusion, the engineered strain and the strategies developed in the present study can be used as the basis for future industrial applications of extremophilic microorganisms.

## Figures and Tables

**Table 1:** Strains and plasmids used in the present study.

	Description	Reference
Strains		
*D. radiodurans* R1	Wild type (ATCC13939)	ATCC
*D. radiodurans* DX1	Wild-type harboring pAM104 plasmid	This study
*D. radiodurans* DX2	Integration of *dxs* and *crtB* genes with the *loxP* scar sequence into the *D. radiodurans* R1 chromosome (CP1)	This study
*E. coli* DH5α	Host for recombinant plasmid construction	Lab stock
Plasmids		
pTOP Blunt V2	TA cloning vector	Enzynomics
pRADZ3	*E. coli*–*D. radiodurans* shuttle vector carrying the *groE* promoter	[25]
pAM1	Derivative of pKatAPH3 containing a *lox66-km^r^-lox71* cassette	[25]
pAM2	Derivative of p13840 containing P*_groE_*-cre-P*_groE_*-*cm^r^*	[25]
pAM41	Derivative of pRADZ3 containing *crtB*	[12]
pAM73	Derivative of pRADZ3 containing *dxs*	[12]
pAM104	Derivative of pRADZ3 containing *dxs* and *crtB*	[12]
Pdrc6-crtB	pTOP Blunt V2 containing two homology arms (the partial sequences of *drC0006* and *drc0007*) and P*_groE_*-*crtB*- *lox66-km^r^-lox71*	This study
Pdrc4-dxs	pAM1 containing two homology arms (the partial sequences of *drC0004* and *drc0005*) and P*_groE_*-*dxs*- *lox66-km^r^-lox71*	This study

## Data Availability

All data underlying the results are included as part of the published article and its Supplementary Materials.

## Supplementary Materials

The following are available online at https://www.mdpi.com/2076-2607/9/1/44/s1, Figure S1: Schematic illustration of the deinoxanthin biosynthetic pathway and the metabolic engineering approach to enhancing deinoxanthin production in *D. radiodurans*; Figure S2. Verification of insertion of *dxs* and *crtB* genes in *D. radiodurans* DX2 genome. (A) Diagnostic PCR of *dxs* or *crtB* gene inserted in DX2 strain. (B) Plating assay of DX2 strain on TGY agar plate with or without kanamycin (25 μg/mL); Table S1: Primers used in this study.
