# Supplementary material for: Metabolic Engineering of Extremophilic Bacterium Deinococcus radiodurans for the Production of the Novel Carotenoid Deinoxanthin
Source: Microorganisms. 2020 Dec 25;9(1):44. doi: 10.3390/microorganisms9010044 (PMC7823818; doi:10.3390/microorganisms9010044)
Supplement: Supplementary file 1 [file microorganisms-09-00044-s001.pdf]

Table S1. Primers used in this study

Primer	Sequence (5′ to 3′)[1]	Restriction enzyme
drc06-1	ttacctcactgtgccaagca	
drc06-2	atgcgcaaatcagaccgtgcaggccttgacgacagccacgcactct	*Stu*I
drc07-3	agagtgcgtggctgtcgtcaaggcctgcacggtctgatttgcgcat	*Stu*I
drc07-4	gatgagacatttcctgtgta	
groE-F1	ctatggtacctcggcttggaagcacgtatt	*Kpn*I
groE-F2	ctataggccttcggcttggaagcacgtatt	*Stu*I
crtB-R1	ctatgatatctcagccgtggaccgcgccca	*EcoR*V
crtB-R2	taacaggccttaccgttcgtataatgtatg	*Stu*I
diadrc06-F	aatgggtatacctcagaacg	
diadrc06-R	caggcaattactgctccaag	
drc04-1	cgtaggtaccgtgtggtaccacgccgatca	*Kpn*I
drc04-2	ccatctcgagctcgctgcagcgcaatcgagttcgtgcgaa	*Xho*I, *Pst*I
drc05-1	ccatggatccactctcacgtcaggccattt	*BamH*I
drc05-2	ctcggcatgctgcttggcacagtgaggtaa	*Sph*I
dxs-F1	ctcgctgcagtcggcttggaagcacgtatt	*Pst*I
dxs-R1	ctcggatatcctacacctcaatcggcacgt	*EcoR*V
diadrc04-F	tccgaaagtacagtccgcac	
diadrc04-R	agcgaggtagaacgaatcgt	

[1] Restriction enzyme site is underlined in the sequence

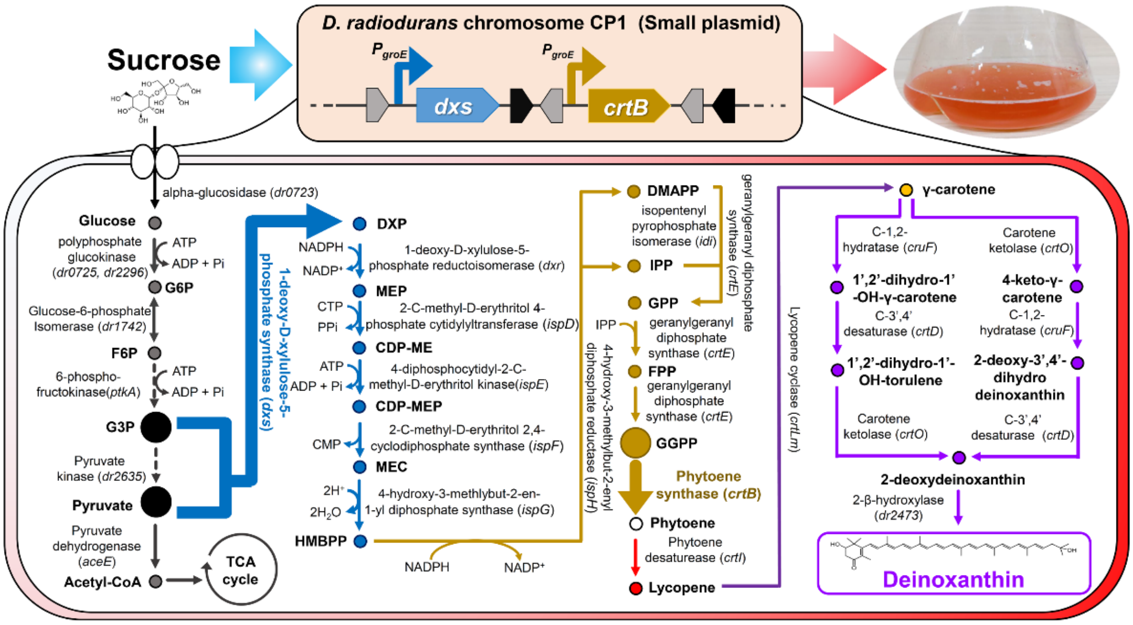

Figure S1. Schematic illustration of the deinoxanthin biosynthetic pathway and the metabolic engineering approach to enhancing deinoxanthin production in *D. radiodurans*. The thick arrows and large circles indicate the increased flux arising from *dxs* and *crtB* overexpression and rate-limiting steps in carotenoid production in *D. radiodurans*. The dashed lines indicate multiple steps in the corresponding metabolic pathway. Intermediate precursors in the metabolic pathway: G6P, glucose 6-phosphate; F6P, fructose 6-phosphate; G3P, glyceraldehyde 3-phosphate; DXP, 1-deoxy-D-xylulose 5-phosphate; MEP,

2-C-methyl-D-erythritol 4-phosphate; CDP-ME, 4-(cytidine 5′-diphospho)-2-C-methyl-D-erythritol; CDP-MEP, 2-phospho-4-(cytidine 5′-diphospho)-2-C-methyl-D-erythritol; MEC, 2-C-methyl-D-erythritol 2,4-cyclodiphosphate; HMBPP, (E)-4-hydroxy-3-methylbut-2-en-1-yl diphosphate; DMAPP, dimethylallyl diphosphate; IPP, isopentenyl diphosphate; GPP, geranyl diphosphate; FPP, farnesyl diphosphate; GGPP, geranylgeranyl diphosphate.
